# The Spanish version of Occupational Balance Questionnaire: psychometric properties and normative data in a representative sample of adults

**DOI:** 10.1080/07853890.2022.2145016

**Published:** 2022-11-11

**Authors:** Paula Peral-Gómez, Cristina Espinosa-Sempere, Eva M. Navarrete-Muñoz, Miriam Hurtado-Pomares, Iris Juárez-Leal, Desireé Valera-Gran, Alicia Sánchez-Pérez

**Affiliations:** a Grupo de Investigación en Terapia Ocupacional (InTeO), Miguel Hernández University, Alicante, Spain; b Department of Surgery and Pathology, Miguel Hernández University, Alicante, Spain; c Alicante Institute for Health and Biomedical Research (ISABIAL-FISABIO Foundation), Alicante, Spain

**Keywords:** Occupational balance, Occupational Balance Questionnaire, psychometric properties, population normative data

## Abstract

**Background:**

The Occupational Balance Questionnaire (OBQ) is an instrument that assesses occupational balance (OB). It has been transculturality adapted and validated in different countries, showing adequate psychometric properties. To date, no general population-based cut-off points for OB have been developed.

**Objective:**

To assess the psychometric proprieties of the Spanish version OBQ (OBQ-E) and to estimate reference norms and the cut-off for OBQ-E in a representative sample of Spanish adults.

**Materials and methods:**

A total of 797 adults were included in this validity study. Internal consistency, intra and test-retest reliability of OBQ-E were examined. To obtain the convergent validity and the divergent validity, the Satisfaction with Life Scale (SLS) and the Hospital Anxiety and Depression Scale (HAD) were used respectively, compared with OBQ-E. To determine extreme and moderate disturbed OB stratified by age, sex, and educational level were used the 5 and 15% percentiles of OBQ-E.

**Results:**

The OBQ-E showed good internal consistency (*α*-Cronbach = 0.87), intraclass reliability (ICC = 0.87), and test-retest reliability (rho = 0.83). Convergent (SLS) and divergent (HAD) validity were moderate (rho = 0.39 and rho = −0.46, respectively). The lowest extreme disturbed OB cut-off point in men (17.2) and in women (24) appeared at the primary education level, under 40 years of age (men) and 40–65 years of age (women).

**Conclusions:**

The OBQ-E presents adequate psychometric properties, and its normative data can be used as a reference to assess and monitor the occupational balance in the general Spanish population.

**KEY MESSAGES**
- The ‘Occupational Balance Questionnaire’ (OBQ), stands out as a specific measure of the concept of Occupational Balance, considered as satisfaction with the number and variation of occupations in which the person participates.
- The OBQ is a short and simple instrument that can be a useful tool for use in population-based and epidemiological studies to monitor OB and explore the associated factors or implications of disturbed OB.
- The Spanish version of the OBQ (OBQ-E) seems to be a reliable and valid questionnaire to assess the perception of balance between occupations, related to health and well-being in the Spanish adult population.

## Introduction

Occupational balance (OB) refers to an individual’s subjective experience of having the right amount and variation of occupations within their occupational pattern [1]. Although research on OB is scarce, several studies have shown that higher OB is associated with better-perceived health in the general population [2–4], people with rheumatic disease [5,6], and people on sick leave [7,8], better mental health in people with arthritis [6]; and better quality of life, higher perceived well-being, higher life satisfaction and lower stress in the general population [3,4,9–13].

OB has been described in different ways, leading to the development of various tools for its measurement [14,15]. For example, considering OB as a harmonic combination between different occupations [15] we can find instruments, such as the ‘Satisfaction with Daily Occupations and Balance’ [16] and the ‘Occupational Balance-Questionnaire’ [17]. However, these tools include the assessment of other issues, such as the level of participation in an activity [16] or the adaptation of activities to living conditions and psychophysical indicators of stress [17]. By contrast, the ‘Occupational Balance Questionnaire’ (OBQ) [18], stands out for specifically and simply measuring the concept of OB, considered as the satisfaction with the number and variation of occupations. Thus, OBQ does not focus on measuring only one aspect of OB (e.g. amount of time spent in occupations, perception of meaningful occupations, or balance between specific occupations) but considers different perspectives (types of occupations, meaningfulness in the occupations, time spent, and perceived satisfaction) linked to the concept. Moreover, this approach bridges individual and cultural differences related to the value placed on different occupations and time use [18].

To date, this instrument has been translated and cross-culturally adapted into Norwegian [19], English [20], Turkish [21], Spanish [22], and Arabic [23]. Likewise, its psychometric properties have been evaluated in healthy populations [18,22,24–26] and in populations with different clinical characteristics [27,28], showing good content validity and reliability. However, to date, they have not been developed cut-off points to classify individuals according to their degree of OB in a representative sample of the population.

Hence, the OBQ is a short and simple instrument that has shown good psychometric properties in other countries. It can be a useful tool for use in the population and epidemiological studies to monitor OB and explore the associated factors or implications of disturbed OB. As far as we know, the OBQ-E has not been previously validated in a representative sample of the Spanish adult population. Therefore, the aim of the present study was to assess the psychometric proprieties of the Spanish version OBQ (OBQ-E) and to determine cut-off points to classify individuals according to age, sex, and education level for different degrees of OB in a representative sample of Spanish adults.

## Method

### Research design and participants

This validity study is part of the NOR + Project (http://inteo.edu.umh.es/en/normas/) whose aim is to evaluate the psychometric properties and establish normative data in a representative sample of Spanish adults of several assessment instruments. The research was approved by the Ethics Committee of the Miguel Hernández University (reference DCP.PPG.02.17) and all participants gave written informed consent. A unique identification number will be used to anonymize confidential personal information on each participant. The principal investigator (P.P.-G.) was responsible for ensuring appropriate data management and storage.

Recruitment was conducted between January 2017 and April 2019 using non-probability snowball sampling. Participants (*n* = 801) were selected considering sex, age, and educational level similar to that of the total Spanish adult general population, using population data published by the National Institute of Statistics [29]. In the study, were not included institutionalizes people, <20 years old, non-Spanish-speaking, non-permanent residents in Spain, with a history of central nervous system disease with possible neuropsychological involvement, with perceptual, visual, or auditory alterations that limited the performance of the tests, with a history of serious psychiatric illness or a history of alcohol and drug abuse, and with any cognitive alteration that could alter their participation. To exclude cognitive impairment, a score of ≥28 points on the Mini-Mental State Examination test was used as a criterion [30]. Finally, after deleting the subjects who had missing information in the OBQ-E, 797 participants were included in this study (99%). The sample was recruited from cultural and neighborhood associations, health centers, and other civic centers. Interested participants were screened face to face and if they met the eligibility criteria, the person completed the full assessment after signing the informed consent form.

### Measures

Data collection was carried out by research assistants trained in the administration of the measures. The OBQ-E and all the other neuropsychological tests were administered in a one-on-one session by only one research assistant.

### Sociodemographic information

Information of age, sex, school level, place of residence, marital status, employment status, dependent children, and native language were collected using an *ad hoc* questionnaire.

### Occupational balance

OB was measured using the Spanish-adapted version of the OBQ (OBQ-E) [22]. The OBQ-E is a brief tool of 13 items scored on a 6-point Likert-type response scale, ranging from completely disagree (score 0) to completely agree (score 5), with the total score ranging from 0 to 65, where a higher score indicates greater OB.

### Anxiety and depression symptoms

The presence of anxiety and depression symptoms was assessed with the Spanish version of the Hospital Anxiety and Depression Scale (HAD) [31] of 14 items (seven in the anxiety subscale and seven in the depression subscale), whose scores range from 0 to 42 points, where higher values indicate greater anxiety and depression.

### Life satisfaction

Life satisfaction was measured through the Spanish version of the Satisfaction with Life Scale (SLS) [32,33], composed of five statements to be scored according to the degree of agreement, using a 7-point Likert-type scale (from 1, strongly disagree; to 7, strongly agree), which score ranges from 5 to 35, with higher scores indicating greater satisfaction.

### Data analysis

All statistical analyses were performed with R 4.0.2 statistical software (R Foundation for Statistical Computing, Vienna, Austria; http://www.r-project.org). Bilateral statistical tests were applied, setting statistical significance at 0.05.

To describe the characteristics of the participants, frequencies presented as *n* and % were used for categorical variables, and median and interquartile range (IQR) for quantitative variables due to variables in our sample were not normally distributed. The Kolmogorov–Smirnov test with the Liliefords correction was used to check the normality of the quantitative variables.

Internal consistency was assessed with Cronbach’s alpha test, establishing ≥0.7 as an adequate value [34].To assess intra-class and test-retest reliability, the OBQ-E was administered to a subsample twice over a period between 4 and 6 weeks. The intraclass correlation coefficient (ICC) was calculated for intraclass reliability and Spearman’s correlation coefficient to measure the stability of the measures (test-retest reliability). A coefficient ≥0.75 was considered a good ICC [35], and a coefficient ≥0.5 was a strong Spearman correlation [36].

The convergent validity between OBQ-E and SLS and the divergent validity between OBQ-E and HAD were assessed with Spearman’s coefficient correlations. We used the SLS as the gold standard due to there is no gold standard in Spain and life satisfaction and OB are considered related constructs.

We described the OBQ-E and established extreme and moderate disturbed OB based on the 5th and 15th percentiles of the distribution stratified by age (<40, 40–65, >65 years), sex (male and female), and educational level group (primary, secondary, university).

## Results

The sociodemographic characteristics of the participants was shown in Table 1. The median age was 46 years (IQR = 32; 58), 54.5% were female, and 36.8% had more than a high school education.

**Table 1.** Demographic and clinical characteristics of the study participants (*n* = 797).

Age, median (IR)	46 (32; 58)
Gender, *n* (%)	
Female	434 (54.5)
Male	363 (45.5)
Education level, *n* (%)	
Uneducated or primary	287 (36)
Secondary	217 (27.2)
Higher education	293 (36.8)
Marital status, *n* (%)	
Without partner	196 (24.6)
Married or living with partner	506 (63.5)
Separated or divorced	57 (7.2)
Widowed	38 (4.8)
Working status, *n* (%)	
Working	481 (60.4)
Retired	141 (17.7)
No working	65 (8.2)
Housewife	51 (6.4)
Student	58 (7.3)
Dependent children, *n* (%)	
Yes	343 (43.3)
No	449 (56.7)

Table 2 presents the results of the internal consistency and reliability of the OBQ-E in a representative sample of Spanish adults. The OBQ-E showed good internal consistency (Cronbach’s *α* = 0.87), and no changes were observed after item-by-item elimination. Intraclass and test-retests reliability for the total OBQ-E were good (ICC = 0.87; rho = 0.83, respectively).

**Table 2.** Reliability of the OBQ-E in general population.

	Internal consistency (*n* = 797)	Intra-class reliability (*n* = 205)	Test-retest reliability (*n* = 205)
	*α* ^a^	ICC	*r_s_*
OBQ-E	0.87	0.87*	0.83*
Item 1	0.86	0.67*	0.67*
Item 2	0.86	0.63*	0.57*
Item 3	0.86	0.69*	0.71*
Item 4	0.85	0.64*	0.64*
Item 5	0.86	0.62*	0.62*
Item 6	0.88	0.58*	0.49*
Item 7	0.86	0.68*	0.68*
Item 8	0.85	0.63*	0.65*
Item 9	0.85	0.69*	0.71*
Item 10	0.85	0.67*	0.66*
Item 11	0.86	0.43*	0.45*
Item 12	0.86	0.58*	0.58*
Item 13	0.86	0.76*	0.76*

OBQ-E: Spanish version of Occupational Balance Questionnaire; *α*^a^: Cronbach’s alpha; ICC: intraclass correlation coefficient; *r_s_*: Spearman coefficient.

* *p* < 0.001.

The Spearman’s correlation coefficient between OBQ-E and SLS was 0.39 (convergent validity) while the Spearman’s correlation coefficient between OBQ-E and HAD was −0.46 (divergent validity) (Table 3).

**Table 3.** Validity measures: correlations between the performance of OBQ-E and the other test in general population (*n* = 797).

	Total
Satisfaction with Life Scale	0.39*
Hospital Anxiety and Depression Scale	
Total HAD	−0.46*
Anxiety HAD	−0.38*
Depression HAD	−0.40*

OBQ-E: Spanish version of Occupational Balance Questionnaire.

*p < 0.001.

The description of OBQ and cut-off points to moderate and extremely disturbed OB according to age group, sex, and educational level was shown in Table 4. The cut-off points for extreme (percentile 5th) and moderate (percentile 15th) disturbed OB were different between age, sex, and educational level, we observed a high cut-off in the >65 group of age, in men, and in the university. For extremely disturbed OB, in women <40 years, we observed cut-off points of 31, 24.6 and 27.5 for ≤ primary, secondary, and ≥ university educational level while in men <40 years, we observed cut-off points of 17.2, 19.1 and 26.9 for ≤ primary, secondary, and ≥ university educational level. In contrast, in women >65 years, we observed cut-off points of 29.4, 34.4, and 36.3 for ≤ primary, secondary, and ≥ university educational level while in men >65 years, we observed cut-off points of 38.8, 40.2, and 44.3 for ≤ primary, secondary, and ≥ university educational level.

**Table 4.** Normative data of OBQ-E in Spanish general population.

Age		Women			Men		
		Level of education			Level of education		
		≤Primary	Secondary	University	≤Primary	Secondary	University
<40	*N*	21	47	91	20	62	59
	Mean (*SD*)	43	40.9	42.7	39.4	40.3	41.8
	Median (IQR)	39	41	43	40.5	41	43
	Percentile 5th	31	24.6	27.5	17.2	19.1	26.9
	Percentile 15th	34	28.9	35	30.5	30	31
	Ceiling effect (*n*)	1	0	1	0	0	0
	Ground effect (*n*)	0	0	0	0	0	0
40–65	*N*	86	44	64	67	45	55
	Mean (*SD*)	43	41.2	39.3	42.7	42.5	45.9
	Median (IQR)	43	41	40.5	43	42	48
	Percentile 5th	24	28.1	27.1	28.9	30.2	27.8
	Percentile 15th	31	32	30.4	33	33	36.4
	Ceiling effect (*n*)	2	0	0	0	1	0
	Ground effect (*n*)	0	0	0	0	0	0
>65	*N*	55	12	14	38	7	10
	Mean (*SD*)	47.5	52.1	48.7	48	48.7	51.6
	Median (IQR)	47	54.5	50	47	46	51
	Percentile 5th	29.4	34.4	36.3	38.8	40.2	41.2
	Percentile 15th	36	43.8	38.9	40.5	42.6	44.3
	Ceiling effect (*n*)	1	1	0	1	0	0
	Ground effect (*n*)	0	0	0	0	0	0

OBQ-E: Spanish version of Occupational Balance Questionnaire; *SD*: standard deviation; IQR: interquartile range.

## Discussion

This study shows that the OBQ-E is a reliable and valid instrument for assessing OB in a representative Spanish population. The instrument displays good values in internal consistency, intra-class reliability, and test-retest reliability, moderate convergent validity with the SLS, and moderate divergent validity (in the negative sense) with the HAD. In addition, we provided a cut-off point according to age, sex, and educational level to classify people in extreme and moderate disturbed OB based on the 5th and 15th percentiles of the distribution. Thus, the lowest cut-off point for extremely disturbed OB in men and in women appears at the level of primary education, in those under 40 years of age (men) and between 40 and 65 years of age (women).

The OBQ-E presents good internal consistency keeping in line with the results obtained in the original Swedish version [18] and the English version [20]. The intra-class reliability of the Spanish version is good without being able to establish comparisons, since to our knowledge, this is the first study to evaluate this psychometric characteristic. Furthermore, the test-retest reliability of the OBQ-E is strong, in line with the original Swedish version. Also, in our study, oscillations in intra-class reliability and test-retest reliability were found when assessed on the different items. These data suggest that this tool should be used as a whole instrument.

A moderate convergent validity has been found between the OBQ-E and the SLS in our study like previous findings of the Swedish questionnaire [4]. In this sense, it should be considered that there is no gold standard for assessing OB, and two questionnaires assessed closely concepts but different constructs. As for the divergent validity between the OBQ-E and the HAD, a moderate negative correlation was found. This result could not be confirmed with previous studies because they did not report this information.

This is the first study that determines cut-off points of OBQ to classify extreme and moderate disturbed OB based on 5th and 15th percentiles according to age, sex, and educational level groups. We observed the highest cut-point to extreme disturbed OB (5% percentile) was shown in men with university educational level while the lowest cut-point we observed in men with < primary educational level. This seems to show that men in this age group tend to have a greater disturbed balance between occupations the lower their level of education is. Although, to our knowledge, there is no other research to date that includes such detailed characteristics with which to corroborate this assertion.

On the other hand, women present the lowest value in the age range of 40–65 years and primary education. Considering the relevance of occupations linked to work and productivity in this period, perhaps the perception of imbalance is related to the characteristics of their employment. It seems that women suffer greater job precariousness, occupational segregation, concentration in certain activities, and worse wages, aspects that are increased with lower levels of studies [37].

Finally, it can be seen that in both sexes the general trend is that the perception of OB improves with age. This could be due to the experience accumulated over the years that comes with knowing how to organize oneself better to meet the expectations and needs of the roles. In this line, the highest value is observed in the group over 65 years of age with university studies in both sexes, being higher in men than in women.

Studies indicate that the two main factors affecting retirement are loss of purchasing power and loss of social function. This could explain why people over 65 with a high level of education have better OB. Of the two factors mentioned, loss of purchasing power affects them to a lesser extent [38]. We think that economic stability, in addition to the experience associated with age, may have an impact on a better organization of their occupations. In addition, the differences found according to sex at this stage may be due to gender roles and the feminization of care that still persists in our country [39]. This implies that, once retirement age is reached, men’s occupational demands associated with work disappear, and they are engaged in care management tasks (not direct care). Meanwhile, women’s demands are partially reduced or transformed, as they maintain occupations associated with household chores and active care of others [40].

This study has several limitations. Firstly, due to its magnitude, different evaluators participated in data collection, which could lead to information bias. However, the evaluators received a training course beforehand and were monitored throughout the duration of the study to ensure correct compliance with the methodology. In addition, the test-retest evaluations of each participant were carried out by the same evaluator to avoid interference. Second, the time interval between such test-retest evaluations was a maximum of 6 weeks, when in other research the established time was 1 week [18]. This may be the reason for the differences in test-retest reliability found between the Swedish version and the Spanish version. However, although the usual time interval between a test and its retest is 2–4 weeks [41], several authors consider longer time periods whenever the context and circumstances are similar on both tests [41,42]. In this study, the extension of the deadline was due to the organizational needs of the evaluators who interviewed participants from different points of the Spanish territory but always performed the tests in similar circumstances. Finally, the study of convergent validity has been done using the SLS, since life satisfaction is related to OB, although they are different constructs. However, it should be noted that there is no gold standard tool for the assessment of OB.

Likewise, this study has several strengths. It is the first investigation that has tested the psychometric properties and established cut-off points in the general population. Thus, it provides novel data and may serve as a basis for future OB research in our context. Also noteworthy is the achieved sample size (*n* = 797), which strikingly exceeds the number of participants in previous studies of psychometric property assessments of the OBQ, with samples ranging from 67 to 217 participants [4,18,20–22]. The large sample size has made it possible to present data of this kind for the first time.

The OBQ-E and the cut-off points provide information on the perception of balance between occupations in the general Spanish population, which will help to set therapeutic objectives in case of disturbed OB. In addition, they serve to measure the changes obtained after the intervention, being a useful resource both in the clinical and research settings. In light of the results obtained, as future lines of research, it would be appropriate to carry out population and epidemiological studies to monitor OB and explore the factors associated with it in detail (such as the influence of work activity and income level) or the implications derived from an imbalance in occupations.

## Conclusions

The OBQ-E is a tool with excellent internal consistency and reliability and moderate concurrent and divergent validity for use in the general Spanish population.

The OBQ-E can be a very useful instrument for both clinical practice and research, and the cut-off points to detect extreme and moderate disturbed OB. I can be used in future studies. Furthermore, it seems to have great potential as a tool for monitoring OB in population and epidemiological studies, covering a growing need in current research focused on OB.

## Data Availability

Datasets generated or analyzed during this study are not publicly available due to ethical approval constraints involving patient data and anonymity but are available from the corresponding author upon email request.
